# Early Contact Stage of Apoptosis: Its Morphological Features and Function

**DOI:** 10.1100/tsw.2006.299

**Published:** 2006-12-28

**Authors:** Etheri Mikadze, Teo Mamatsashvili

**Affiliations:** Laboratory of Developmental Biology, Iv. Javakhishvili Tbilisi State University, Tbilisi, Georgia

**Keywords:** apoptosis, contact stage, agglutination of ribosomes, hepatoblast, hepatocyte, mitotic cell, cycloheximide

## Abstract

Apoptosis has been a biological phenomenon of intense interest for 20 years, but the earlier morphological features of apoptosis have not been determined hitherto. Using the methods of semi- and ultrathin sections, the livers of intact embryos and young rats have been studied under the effect of cycloheximide to determine morphological features of an early stage of apoptosis. It is discovered that both in hepatoblasts and hepatocytes, apoptosis, besides the well-known stages, also includes an early contact stage, distinguishing features of which are agglutination of bound ribosomes (breaking of translation), elimination of the nucleolus, reduction of free polysomes (and in hepatocytes, reduction of cisterns of rough endoplasmic reticulum), formation of cytoplasmic excrescences, and cell shape changes. The early stage of apoptosis is characterized by close contact with neighboring cells. At a certain phase of the contact stage of apoptosis, the nucleolus reappears in the nucleus and the number of free polysomes in the cytoplasm increases, which suggests the renewal of synthesis of new RNA and proteins. Close contact of differentiating and mitotic hepatoblasts with apoptotic cells indicates a certain functional relationship between these cells that is realized not only by micropinocytosis, but through gap junctions as well. We assume that the apoptotic cell, besides proteolytic products, can contain newly synthesized, low-molecular substances, the relocation of which from apoptotic to neighboring cells may contribute to both functional activity and proliferation of adjacent hepatoblasts and, therefore, the function of apoptosis may not be limited only to the elimination of harmful, damaged, and unwanted cells.

### INTRODUCTION

In 1972, Kerr et al.[1] coined the term "apoptosis" in order to indicate programmed cell death (PCD). Apoptosis is a basic physiological phenomenon that appears to play a complementary, but opposite, role to mitosis in the regulation of cell population, and is characterized by the universal ultrastructural peculiarities common for all types of cells that are fundamentally distinct from those of necrosis. However, the initial morphological events and earlier features of apoptosis had not been identified[1,2].

In mammals, the regulation of apoptosis is realized by two signaling pathways: "extrinsic", through binding of receptor-ligand to the surface of target cell followed by the activation of initiator caspase-8; and "intrinsic", induced by various internal or external stresses that can cause: (1) the damage of mitochondrial membrane permeability (MMP) and release of cytochrome *c* from their matrix, which binds to cytosolic Apaf-1 (apoptosis protease activating factor-1) and ATP, and forms apoptosome, which in its turn recruits procaspase-9[3,4,5,6,7]; (2) the stress of the endoplasmic reticulum (ER) that also can be accompanied by acute $Ca^{2+}$ release. In this case, apoptosis can be realized by the activation of procaspase-12, localized on the cytoplasmic side of the ER[8].

It is well known that apoptosis is easily induced by both different physiological activators and toxins of different type. Cycloheximide (CHX), which is the powerful inhibitor of protein synthesis and apoptosis inductor[9,10,11,12], is also used in the studies dedicated to the translation regulation under the conditions of stress and apoptosis[12,13,14]. Recently, it has been shown that induction of apoptosis is associated with a rapid and substantial inhibition of protein synthesis. The inhibition of protein synthesis is accompanied by the changes of the multiple components that include translation initiation factors, the ribosome and cellular level of mRNA[13,14,15]. These changes cause the ER stress, which has now been recognized as a third subcellular compartment in the control of apoptosis and which, in turn, triggers unfolded protein response (UPR). The UPR leads to the inhibition of global protein synthesis, and the selective transcription and translation of proteins, which commonly occurs during cellular stress and apoptosis[16].

According to the reports of the last 10–20 years, the morphological features of apoptosis described by Kerr, Wyllie, and Currie[1,2] are more often subjected to reconsideration; both the identity of apoptosis and PCD and its morphological universality are considered to be doubtful. Schwartz et al.[17] supposed that some cells can use the mechanism of PCD distinct from apoptosis and, therefore, it is not correct to use the terms "apoptosis" and PCD interchangeably. There is some information in the literature about alternative and nonapoptotic forms of cell death, resulting from their morphological inadequacy with the "gold standard" of apoptosis[18,19,20]. In induced mouse mastocytoma, Harmon[21] revealed the third morphological type of cell death, "dark cell". Turmaine et al.[22] think that during Huntington's disease, neuronal cell death occurs as "dark cell degeneration".

At the same time, there exists an opinion that cell death during which caspases have been blocked often bears little morphological similarity to apoptosis and can even look surprisingly similar to classical necrotic cell death[6], and that the programmed dying cell is sometimes enigmatically devoid of the characteristic morphology of apoptosis[23]. According to Lockshin and Zakeri[24], the period of semantics passed and what we should understand today is (1) that caspase-controlled cell death (apoptosis) is a biological phenomenon of substantial theoretical and clinical importance, (2) that cells also have the option to undergo autophagic deaths, and (3) that we need to understand both the signaling mechanisms and the effector mechanisms for cell death.

Also, there is an idea to create more reliable and sophisticated assays[6,20,23] for the exact detection of apoptotic cells because the existing methods (gel electrophoresis, histochemistry, flow cytometry, TUNEL, detection of mitochondrial transmembrane potential) are not always specific for the identification of apoptotic cells only. Van Cruchten and Van den Broeck[23] think that, at present, the best results are obtained with electron microscopy.

It must be noted that despite the existence of a lot of data about the mechanisms, pathways of realization of apoptosis, and genes responsible for this process, its earlier morphological features have not been determined hitherto.

Therefore, the aim of our investigation was to study the parenchymal cells of the liver, both in intact embryos and in young rats after the impact of cycloheximide, using the methods of semi- and ultrathin sections in order to determine earlier morphological features of apoptosis and dynamics of subcellular changes at the early stage of apoptosis. At the same time, based on the comparative analysis of the morphological changes taking place during apoptosis in the cells of both studied objects, and reasoning from the fact that the cycloheximide is the protein synthesis inhibitor, we also aim to determine the possible stimulus that might trigger apoptosis in the cells of the fetal liver.

## MATERIALS AND METHODS

### Animal Procedures

Animal treatment was performed according to the *Guide for the Care and Use of Laboratory Animals* (National Academy Press, Washington, D.C., 1996). Albino outbred rats were used in the experiments. Five females were placed with one male rat. The day of sperm-positive smears was taken as the first embryonic day (ED). Pregnant rats on ED 15, 17, 19, and 21 were anesthetized with an intraperitoneal injection of Nembutal, according to body weight. The embryos were removed from the uterine tube of pregnant rats under sterile conditions. The liver from the embryos was cut under a stereoscopic microscope.

To induce apoptosis, young male rats (80–100 g) were treated with a sublethal dose of cycloheximide (3.5 µg/g). The liver was removed at 1, 2, 3, and 4 h after the single intraperitoneal injection of cycloheximide.

### Light and Electron Microscopy

Immediately after the removal of the liver, approximately a 1-mm$^3$ tissue slice was fixed in 2.5% glutaraldehyde in 0.1 *M* Na-K-phosphate buffer (pH 7.2). After being osmicated in 1% $OsO_4$ in the same buffer at 4°C, the samples were dehydrated in graded ethanol series and acetone, and embedded in an Epon/Araldite mixture. Sections were cut on a “Reichert Om-U2” Ultracut ultramicrotome. Semithin (1–1.5 µm) sections were stained with 1% toluidine blue and examined in a “Rathenow Row” light microscope. Ultrathin (800–1000 Å) sections were stained with uranyl acetate and lead citrate, and examined in a “TESLA-500BS” electron microscope.

## RESULTS

### Light Microscopy of Semithin Sections of Rat Fetal Liver

The liver tissue of rat embryos of different ages contains a large number of dark cells with an irregular form and polymorphic cytoplasmic excrescences (Fig. 1a–d) that stand out and differ from other types of cell populations: differentiating hepatoblasts (Fig. 1a), small dark hematopoietic cells (Fig. 1b,c), megakaryoblasts (Fig. 1d), and mitotic cells (mitosis both in solitary and in adjoining cells – synchronic mitosis) (Fig. 1b–d). In fetal liver, these irregular cells are randomly diffused and are mainly seen as groups or clusters of two to four cells, which are closely bordering each other and neighboring cells, particularly differentiating hepatoblasts and mitotic cells (Fig. 1a–d). The irregular form and high basophily of these cells indicate destructive processes in them. The morphology of these cells differs sharply from necrosis, the typical feature of which, as is known, is swelling. As during embryological development, massive cell death is genetically programmed[25,26] and is realized by apoptosis[1,2]; we suppose that these dark cellular elements of irregular shape are cells at the early stage of apoptosis, one of the features of which is close contact with adjacent cells. After this stage, the cells separate from the neighboring structures and enter into the first discrete stage of apoptosis[1,2]. The separated apoptotic cells and their polymorphic fragments, apoptotic bodies, are seen more rarely in the fetal liver, which may be caused by their rapid utilization in the embryonic tissue.

During apoptosis, the increase of the degree of condensation and cytoplasmic excrescences condition the significant changes of cell shape, and they become complementary to the form of adjoining structures. Soma of apoptotic cells and their excrescences penetrating into the space between the neighboring cells (Fig. 1a–d) often contain polymorphic vacuoles (Fig. 1a,b).

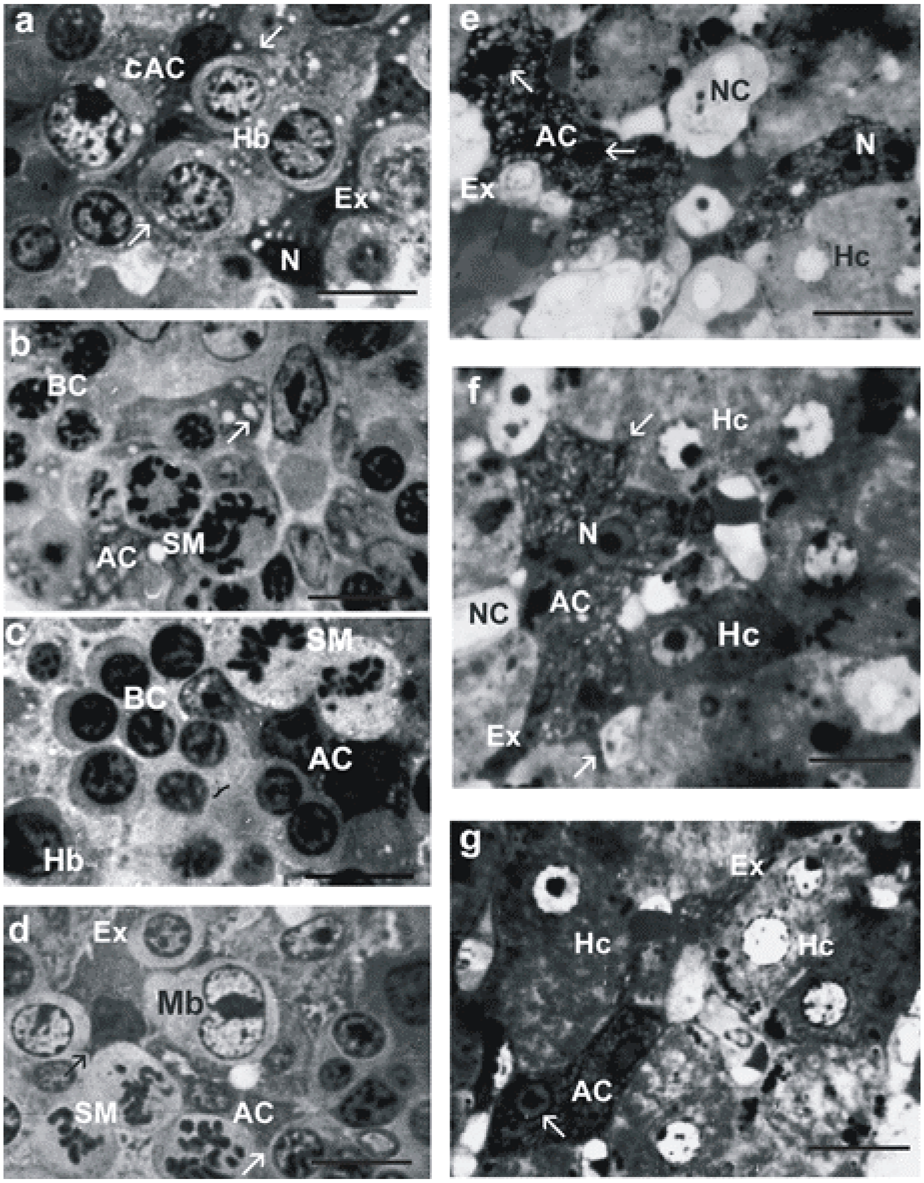

**FIGURE 1.** Apoptotic cells in liver tissue of intact embryos and young rats after cycloheximide administration. (a) Clusters of two to three apoptotic cells (cAC) adjoining hepatoblasts (Hb); condensed nuclei (N) and polymorphic excrescences (Ex, arrows). ED15. (b) Apoptotic cells contain large vacuoles (arrow) adjoining synchronous mitosis (SM). ED19. (c) Group of irregular cells at different levels of contact stage of apoptosis adjoining synchronic mitosis; blood cells (BC). ED17. (d) Soma and excrescences of apoptotic cells surrounding megakaryoblast (Mb) and synchronous mitosis. ED19. (e) Irregular apoptotic cells with dark and condensed nuclei (arrows) adjoining hepatocytes (Hc) and necrotic cells (NC). Two hours after injection of cycloheximide. (f) Binuclear apoptotic hepatocyte with polymorphic excrescences (arrows) adjoining intact hepatocytes; apoptotic hepatocyte with elongated excrescences (Ex). Three hours after injection of cycloheximide. (g) Irregular apoptotic cell adjoining large intact hepatocytes. In nucleus of apoptotic hepatocyte (arrow) possibly new formed nucleolus. Excrescences of apoptotic cells are seen between the hepatocytes. Four hours after injection of cycloheximide. Scale bars 50 μm.

The mitotic and apoptotic cells are arbitrarily distributed in fetal liver and they are not characterized by the focal localization. At the same time, clear regularity is seen in the coarrangement of mitotic and apoptotic cells, and it is expressed in a close contact between these cells (Fig. 1b–d). We suppose that this fact deserves special attention since the information about such a coarrangement of dying and proliferative cells is unknown to us.

### Light Microscopy of Semithin Sections of Young Rat Liver

It is well known that cycloheximide is a powerful inhibitor of protein synthesis on bound polysomes in the cytoplasm[27,28,29] and an inducer of apoptosis as well[9,10,11,12], for it is widely used by researchers. In young rat liver tissue after cycloheximide administration, especially after 3 h of injection, three populations of hepatic cells are seen: irregular dark cells with polymorphic cytoplasmic excrescences, which by configuration and distribution in the tissue are similar to apoptotic hepatoblasts of fetal liver; swollen necrotic cells; and comparatively intact hepatocytes (Fig. 1e–g). After a single injection of cycloheximide, the number of apoptotic hepatocytes increases with the time of the antibiotic influence; in 1, 2, 3, and 4 h after the injection this parameter was, respectively, equal to (per 500 cells) $82 \pm 5$, $175 \pm 6$, $239 \pm 7$, and $149 \pm 4$. In 1 h after cycloheximide treatment in liver tissue, a small number of apoptotic cells is observed. These are most labile and committed-to-death hepatocytes that were first to enter apoptosis. These cells are characterized by a changed shape and small cytoplasmic excrescences. In 2 h after antibiotic injection, the number of apoptotic cells increases at the expense of intact hepatocytes that enter apoptosis in response to inhibition of protein synthesis; the liver tissue contains both more condensed apoptotic hepatocytes with polymorphic cytoplasmic excrescences (Fig. 1e) and cells with insignificant shape changes. In 3 h after cycloheximide administration, the number of apoptotic cells reaches the maximum and they are mainly represented by advanced forms (Fig. 1f), but the degree of nuclear condensation and the size of cytoplasmic excrescences of these cells can be different. Though the number of apoptotic cells decreases in 4 h after cycloheximide injection, advanced and early forms similar to those revealed in 2 h (Fig. 1g) are observed as well. We suppose that such dynamics of qualitative and quantitative changes of apoptotic cells after a single injection of antibiotic indicates both high rates of apoptosis and asynchronous entering of cells into cycloheximide-induced apoptosis. Kerr et al.[1] also noted that individual cells of the same tissue may enter into apoptosis at different times.

The early stage of apoptosis of young rat liver hepatocytes, as well as that of fetal hepatoblasts, is characterized by changes in their shape caused by condensation of the nucleus and formation of polymorphic cytoplasmic excrescences. Both mono- and binuclear hepatocytes are subject to apoptosis (Fig. 1f). Although apoptotic hepatocytes and hepatoblasts are similar by their configuration, there are certain differences between them: the structure of apoptotic hepatocytes is rough and mosaic, the nuclei are more condensed and dark, and their sizes are significantly larger (Fig. 1e–g). These differences are explained by experimental conditions: physiological apoptosis in hepatoblasts of rat embryo and the induced apoptosis in differentiated functionally active hepatocytes of rats.

### Ultrastructural Features of Apoptotic Hepatoblasts

According to the results of the ultrastructural studies, fetal liver of rats of all studied ages contains hepatocytes at different stages of both maturation and destruction. The presence of hepatoblasts in fetal liver at different stages of destruction and the comparison of their ultrastructure with that of intact hepatoblasts contribute to the establishment of peculiarities of subcellular changes characterizing the early stage of apoptosis. At the levels of both light and electron microscopy, the characteristic feature of the early stage of apoptosis is a close contact of apoptotic cells with neighboring ones. This fact allows us to determine the early stage of apoptosis as the contact stage, while the first discrete stage as described by Kerr et al.[1] can be considered as the terminal stage of apoptosis.

The micrographs presented (Figs. 2 and 3) reflect to a certain extent the dynamics of ultrastructural changes that can be observed during the early contact stage of apoptosis when it is initiated in mostly immature parenchymal cells of liver. At the earliest stage of apoptosis in immature cytoplasm, on the background of a

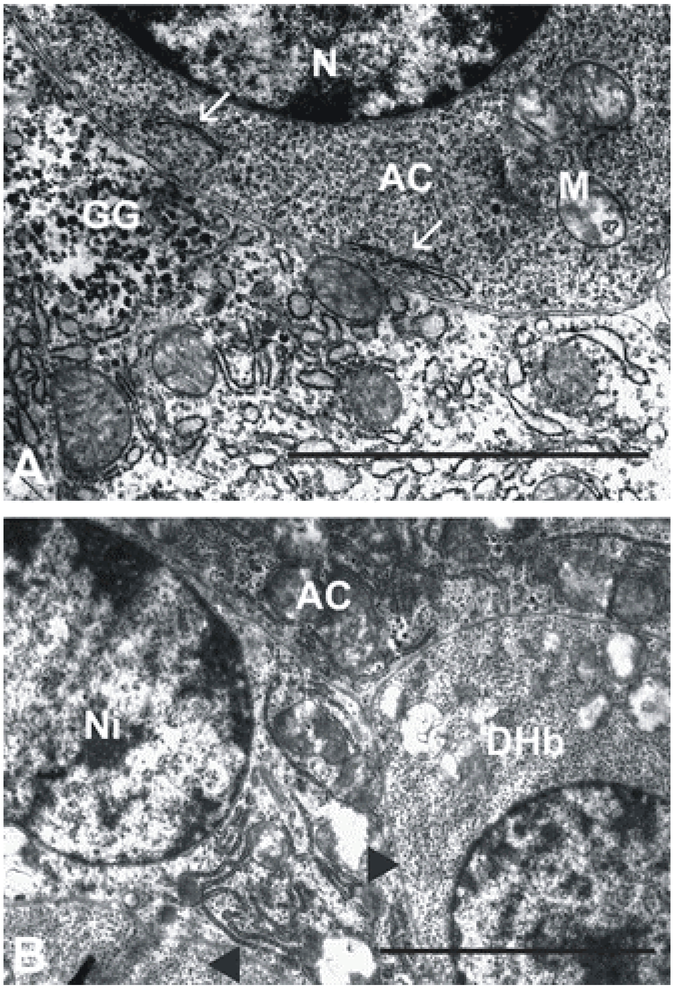

**FIGURE 2.** Peculiarities of ultrastructural changes of hepatoblasts in fetal liver of rat embryos of different ages at the early stage of apoptosis. (A) Hepatoblast (above) at the early stage of apoptosis (AC), in cytoplasm two cisterns with agglutinated ribosomes (arrows); a great number of free polysomes and solitary mitochondria (M). Fragment of cytoplasm of apoptotic hepatoblast (below) containing increased number of cisterns of RER with agglutinated ribosomes, granules of glycogen (GG) and mitochondria. ED15. (B) Spindle-shaped hepatoblast conditioned by formation of small excrescences (arrowheads); in cytoplasm reduction of free ribosomes and increase of cisterns of RER with agglutinated ribosomes are observed; nucleus contains small inactive nucleoli (Ni); in the right differentiating hepatoblast (DHb) contains a great number of free polysomes. ED19. (C) Irregular apoptotic hepatoblast with round nucleus and dissolved nucleolus adjoining differentiating hepatoblasts. Consistency of cytoplasm of apoptotic cell compared with that in B is increased; ultrastructure of nuclei of these cells to some extent is intact. ED19. (D) Apoptotic hepatoblast with small excrescences (black arrows), the cytoplasm of which contains increased number of elongated and flat cisterns with agglutinated ribosomes (white arrow), small mitochondria and remains of Golgi apparatus (GA); their form differs from that presented in C, but the ultrastructure of nuclei is the same; no nucleolus. ED21. (E) Close contact between mitotic (MC) and apoptotic (AC) cells; apoptotic hepatoblast contain nucleus with irregular shape and two nucleoli; expanded perinuclear space (one arrow) and swollen cisterns of RER (double arrows). Degree of basophility is higher at the expense of the increased number of free ribosomes. ED19. (F) Fragments of three irregular apoptotic hepatoblasts surrounding differentiating hepatoblasts. Ultrastructure and form of apoptotic cells are similar to those presented in E. ED17. Scale bars 5 μm.

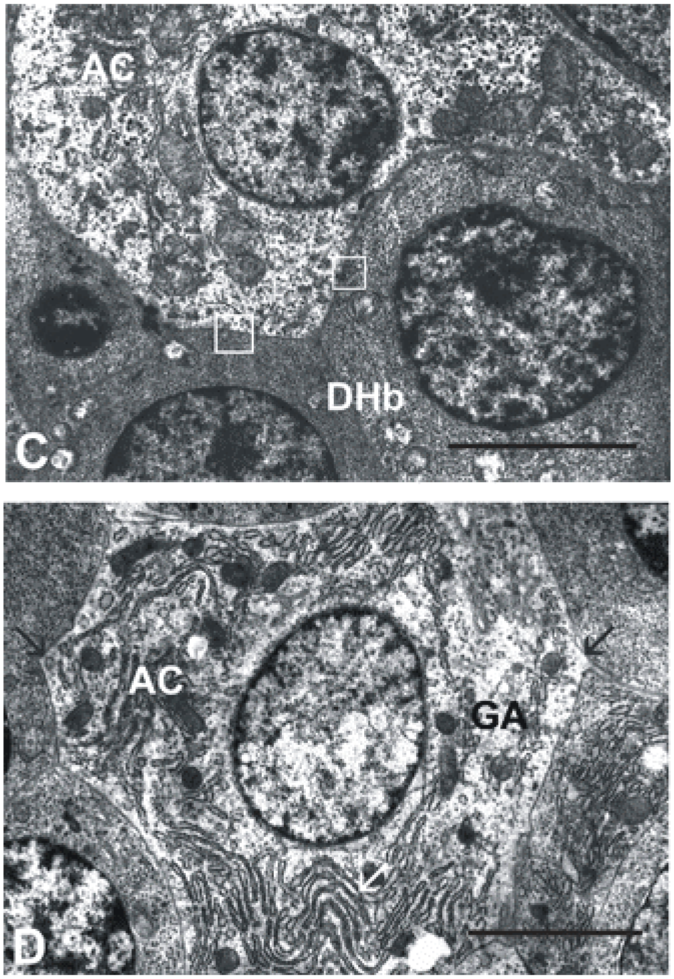

**FIGURE 2C,D**

great number of free ribosomes and polysomes, two cisterns with agglutinated ribosomes are clearly seen (Fig. 2A, arrows). With the advance of apoptosis in the cytoplasm of the hepatoblast (Fig. 2B, left), a significant reduction of free polysomes and an increased number of cisterns of rough endoplasmic reticulum (RER) with agglutinated ribosomes can be observed, and as a result of a small excrescence forming the rounded shape of cell becomes spindle shaped.

The higher the number of cisterns with agglutinated ribosomes in the cytoplasm, the higher the degree of changes in the shape of apoptotic hepatoblasts (Fig. 2C,D). In the nuclei of apoptotic cells, the ultrastructure of which is similar to that of intact hepatoblasts (Fig. 2C,D, right), the nucleoli are not seen (Fig. 2C,D). The absence of nucleoli and the reduction of free ribosomes represent an important event of the early stage of apoptosis.

The hepatoblasts presented reflect changes that take place at the beginning of the early stage of apoptosis. During this time, the cytoplasm of apoptotic hepatoblasts contains few small agranular vesicles, polymorphic mitochondria, sometimes incompletely developed Golgi apparatus, granules of glycogen, solitary dark inclusions, and does not contain either active lysosomes or peroxisomes. Changes at the early stage of apoptosis mainly affect the form of cells and cytoplasmic organelles. At the same time, the ultrastructure of the nucleus remains similar to that of the intact hepatoblast with the exception of the absence of nucleoli.

On a certain phase of contact stage of apoptosis, the nucleolus reappears in the nucleus and the number of free ribosomes in the cytoplasm increases (Fig. 2E,F). The number of polymorphic cisterns of

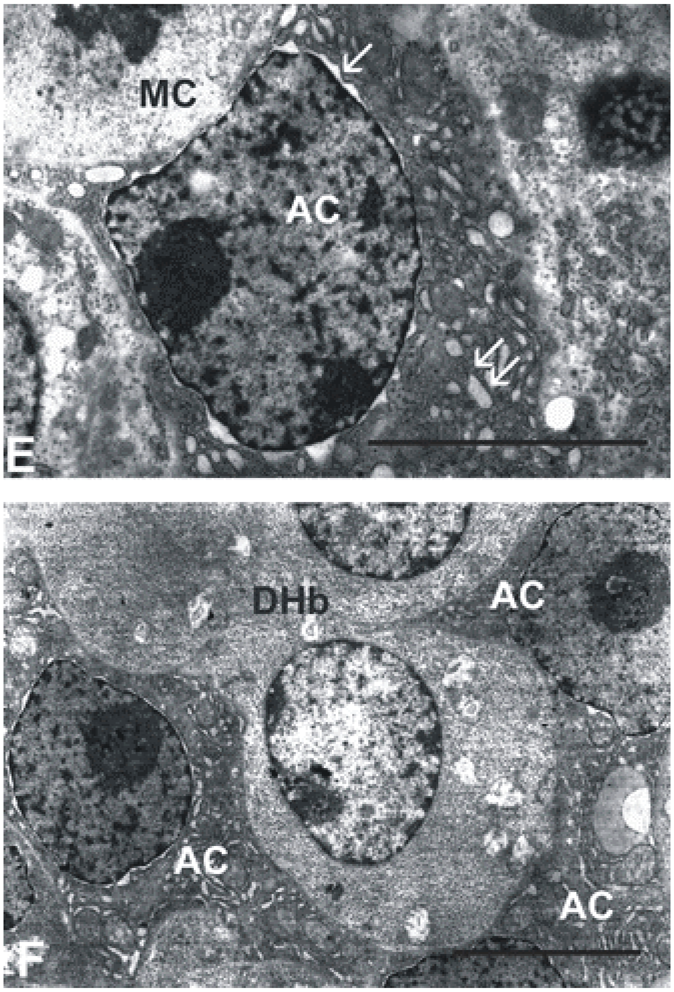

**FIGURE 2E,F**

RER with agglutinated ribosomes increases; they expand and disperse in the whole cytoplasm (Fig. 2E,F). The round nucleus of the hepatoblast gradually shrinks (creating invaginations of different degrees), the perinuclear space expands, and in the karyoplasms, clumps of heterochromatin are observed. The changes in ultrastructure of the nucleus indicate its involvement in the process of apoptosis. Swollen cisterns of RER and expanded perinuclear space are clearly seen in the cytoplasm, the electronic density of which increases at the expense of agglutination of already free polysomes and the degree of condensation of the cell as well. The shape of the cell, excrescences, and sometimes even of the nucleus strictly depends on the shape of neighboring cells, and the form of apoptotic cells becomes complementary to that of compactly adjoining cells (Figs. 2E,F and 3A). Thin cytoplasmic excrescences penetrate between the differentiating and mitotic hepatoblasts (Fig. 3B, arrows). The terminals of excrescences separated from the soma of the cell can be considered as a variety of apoptotic bodies (Fig. 3C).

At the end of the early stage of apoptosis, the intensive swelling of the cisterns of RER and perinuclear space is observed in the cytoplasm of hepatoblast (Fig. 3E). The increase of the degree of the cytoplasm condensation and the splitting of terminals of the cytoplasmic excrescences on the whole cause a decrease in the size of the cell (Fig. 3F). It must be noted that the ultrastructure of most mitochondria of apoptotic cells is virtually undamaged even in the terminals of cytoplasmic excrescences (Fig. 3C). Breakup of the nucleus into fragments with significant chromatin margination, the specific feature of the first discrete stage of apoptosis[1,2], is observed in the apoptotic cell separated from the neighboring ones (Fig. 4A).

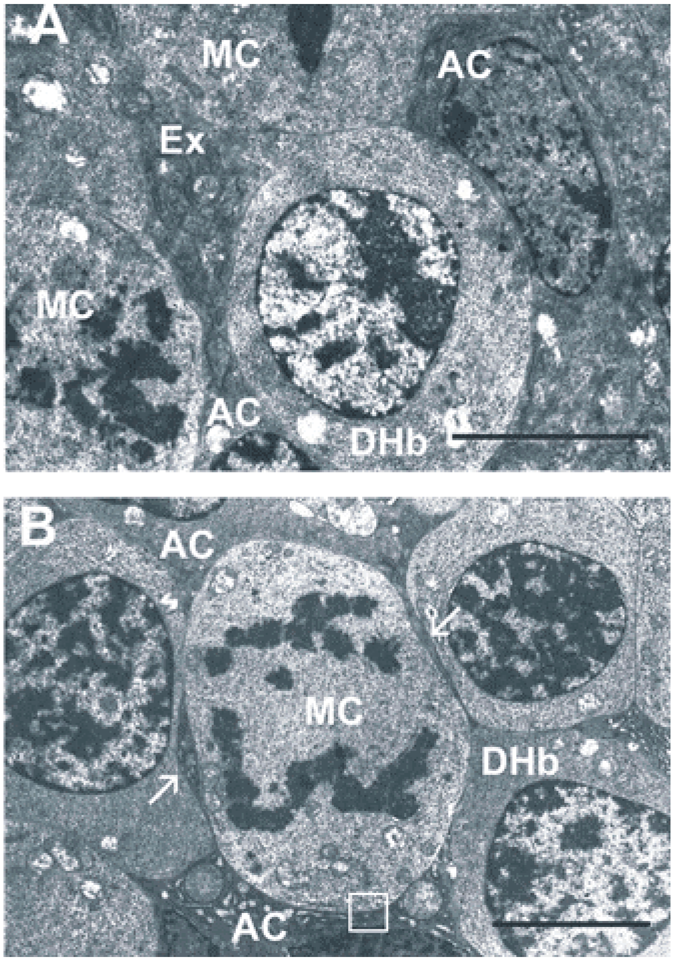

**FIGURE 3.** Peculiarities of ultrastructural changes of hepatoblasts in fetal liver of rat embryos of different ages at the early stage of apoptosis (continuation). (A) Soma and the excrescences (Ex) of apoptotic cells (AC) adjoining mitotic cells (MC) and differentiating hepatoblast (DHb). ED19. (B) Fragments of apoptotic cells at poles of mitotic cell; thin excrescences (arrows) penetrating between mitotic and differentiating hepatoblasts. ED19. (C) Fragment of excrescence of apoptotic cell that can be considered as apoptotic body (AB) in the space between the differentiating hepatoblast and telophase cell (TPh). ED19. (D*) Disruption of integrity of plasma membranes between apoptotic (AC) and adjoining cells (Fig. 2C frame on the right). (D**) Formed and forming micropinocytic vesicles (white arrows) and vesicles (black arrows) between apoptotic (AC) and adjacent calls (Fig. 2C, frame in the center). (D***) Contacts between the cells are presented as three dense staining layers alternating with two light ones (frame in Fig. 3B). (E) Irregular cell with polymorphic cytoplasmic excrescences is characterized by significant expansion of perinuclear space (PnS) and cisterns of RER (arrow). ED19. (F) Decrease of apoptotic cell in size resulted by reduction of cytoplasmic excrescences; in irregular nucleus a large nucleolus is seen. ED19. Scale bars A, B, C, E, and F, 5 μm; D*, 0.1 μm; D** and D***, 0.2 μm.

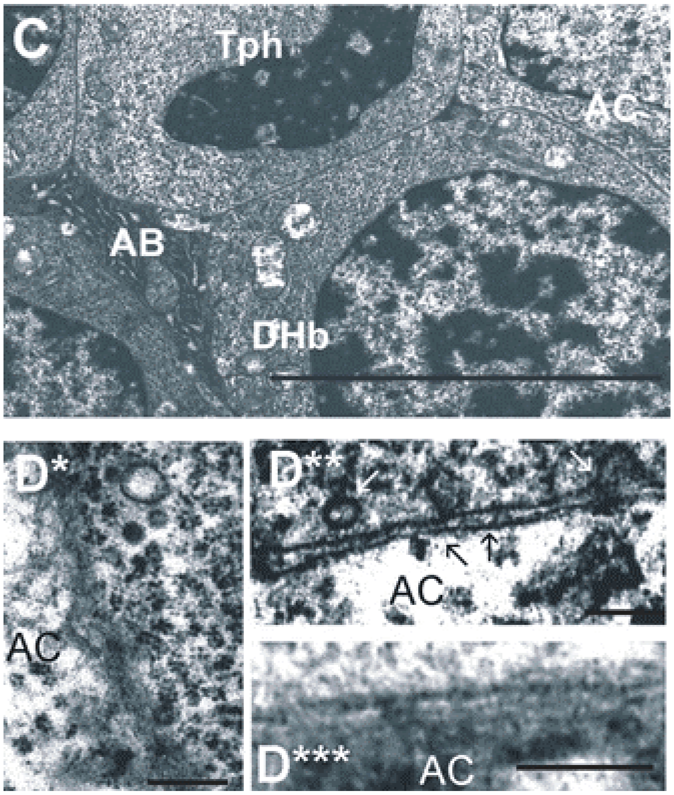

**FIGURE 3C,D**

The ultrastructure of contacts of apoptotic cells with mitotic and differentiating cells deserves special attention. Although at small magnifications the contacts of plasma membrane of apoptotic cells and its excrescences with neighboring cells mainly look like clear lines, at high magnifications of electronic microscopy, it is clearly seen that these contacts are morphologically different even within the limits of the same apoptotic cell. This peculiarity is typical of the early contact stage of apoptosis. The obvious case of the aforesaid can be that presented in the apoptotic cell in Fig. 2C, the contacts of which with two adjacent cells significantly differ by ultrastructure. The aggregate of plasma membranes of apoptotic cell and hepatoblast (below, in the right) is presented as dense diffuse structure (Fig. 3D*), while in contact area beneath the plasma membrane of apoptotic cell with adjoining hepatoblast (below, in the center), both formed and forming micropinocytic vesicles are seen; and in the space between the plasma membranes, vesicles commensurable with intercellular gap in size are observed (Fig. 3D**). When outlines of plasma membranes of apoptotic and adjacent cells are relatively clear, the chains of dark granules are seen on them. Sometimes one can observe the regions where the contacts between the cells are presented as three dense staining layers alternating with two light ones. On dark layers, chains of granules of different size are distributed, as is observed between the apoptotic and mitotic cells (Fig. 3D***, frame in Fig. 3B).

### Ultrastructural Features of Apoptotic Hepatocytes

Ultrastructure of hepatocytes of young rat liver tissue during cycloheximide-induced apoptosis differs from that of apoptotic hepatoblasts of fetal liver. Differentiated hepatocytes of young rat liver are large (15–18 µm) multifunctional cells, rich in organelles. The effect of cycloheximide, an inhibitor of protein synthesis on bound polysomes, first of all is reflected on the ultrastructure of RER, which after 1 h of

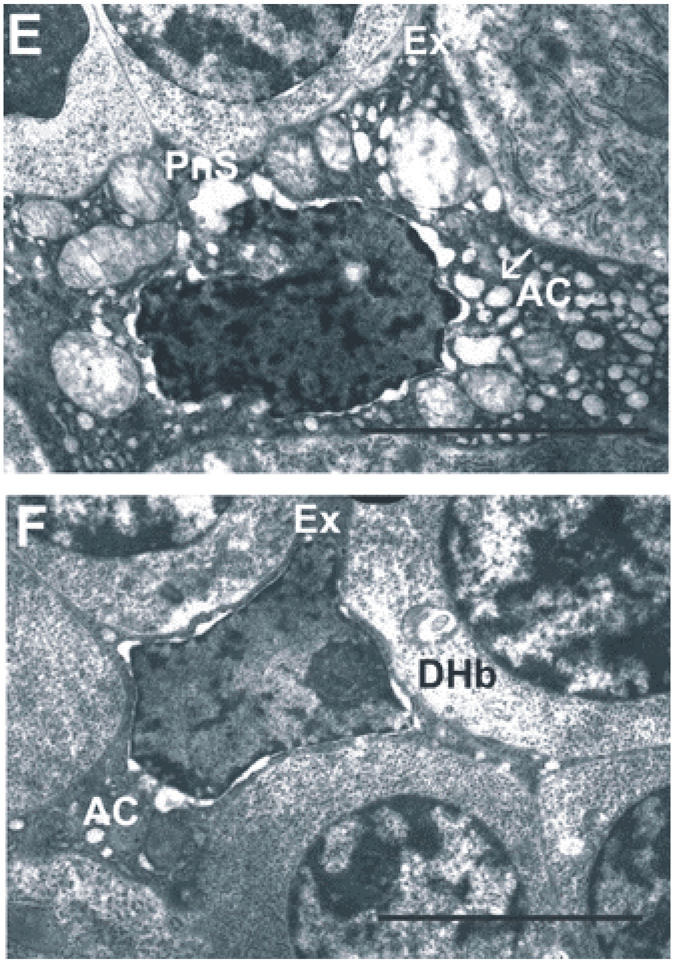

**FIGURE 3E,F**

cycloheximide injection is significantly reduced, and on the residual cisterns of RER the agglutinated polysomes are observed (Fig. 4B). At the same time, as in apoptotic hepatoblasts, the decrease in number of free polysomes is observed. Changes in ultrastructure of hepatocytes after the cycloheximide administration correspond to those described by Todorov[28]. In the cytoplasm of apoptotic hepatocyte electronic-lucent areas, granules of glycogen and small dark mitochondria are observed (Fig. 4B,C). Outlines of large round nuclei are smooth, there is no expansion of perinuclear space, small clamps of condensed chromatin, and sometimes small nucleoli are seen in karyoplasm (Fig. 4C).

In liver tissue after 2 h of cycloheximide injection, both cells with morphological features described above and more advanced forms of apoptotic cells are observed. As a result of gradual reduction and fission of the elements of RER and Golgi apparatus, the empty spaces of different sizes containing the remains of incompletely utilized molecules are formed in the cytoplasm. The cytoplasm of the hepatocyte mainly contains small dark mitochondria with disorganized cristae, small vesicles, and dense inclusions (Fig. 4D). Chromatin margination, which at the early stage of apoptosis is observed just in some interpore areas of nuclear membrane, gradually increases; nucleoli are not seen (Fig. 4D,E).

Cisterns of RER flatten, and, as a result of agglutination of polysomes on their surface, they look like elongated bars (Fig. 4E, arrow). During the early stage of apoptosis, empty areas in the cytoplasm of the hepatocyte disappear, probably as a result of their being filled by organelles remaining in the cell – mitochondria and small vesicles; the cell condenses, forms cytoplasmic excrescences of different sizes (Fig. 4E,F), and becomes electronic dense at the expense of a great number of small dark mitochondria.

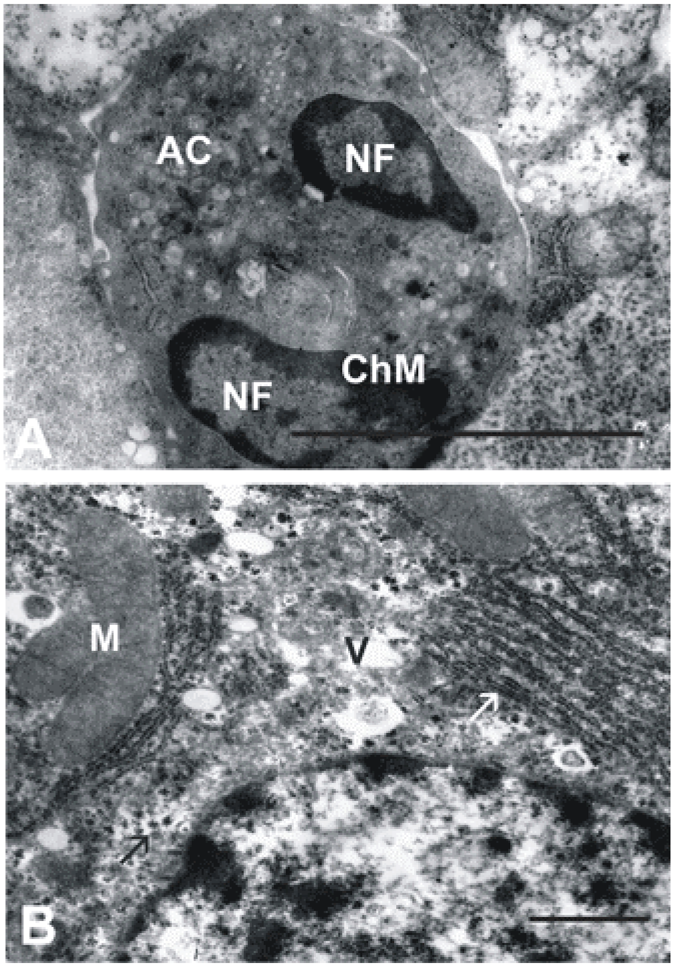

**FIGURE 4.** Apoptotic hepatoblast and ultrastructural changes of hepatocytes of young rats at the early stage of apoptosis. (A) First discrete stage of apoptosis; nuclear fragments (NF) with marginated chromatin (ChM). ED19. (B) Agglutination of ribosomes on cisterns of residual RER (white arrow), dark large mitochondria (M), vacuoles (V), granules of glycogen (black arrow). One hour after injection of cycloheximide. (C) Vacuoles, small dark mitochondria, reduced RER, and round intact nucleus with small nucleolus (Ni) in apoptotic cell (AC) at the early stage of apoptosis. One hour after injection of antibiotic. (D) Apoptotic hepatocyte containing small vesicles, polymorphic mitochondria, and dark inclusions. Two hours after injection of cycloheximide. (E) Excrescence (Ex) of apoptotic hepatocyte penetrating into necrotic cell (NC); cisterns of RER are presented as elongated and flat bars surrounding mitochondria (arrow); nucleus with primary feature of chromatin margination not containing nucleolus. Two hours after injection of cycloheximide. (F) Apoptotic hepatocyte, the cytoplasm of which is transformed into large excrescence (arrowheads) and contains compact mitochondria; nucleolus absent. Three hours after injection. Scale bars A, C–F, 5 µm; B, 1 µm.

In 3 h after of cycloheximide injection, in the cytoplasm of most cells, the number of free ribosomes and polysomes increases (which agglutinate shortly after as well) and large nucleoli are again seen in the nuclei (Fig. 5A), just as in apoptotic hepatoblasts (Fig. 2E,F). During apoptosis, the perinuclear space and cisterns of RER expand (Fig. 5A). The degree of chromatin margination increases, accompanied by elimination of pore structures; as a result, the distance between neighboring pores increases and the perinuclear space expands; such changes were observed by other authors[2] as well.

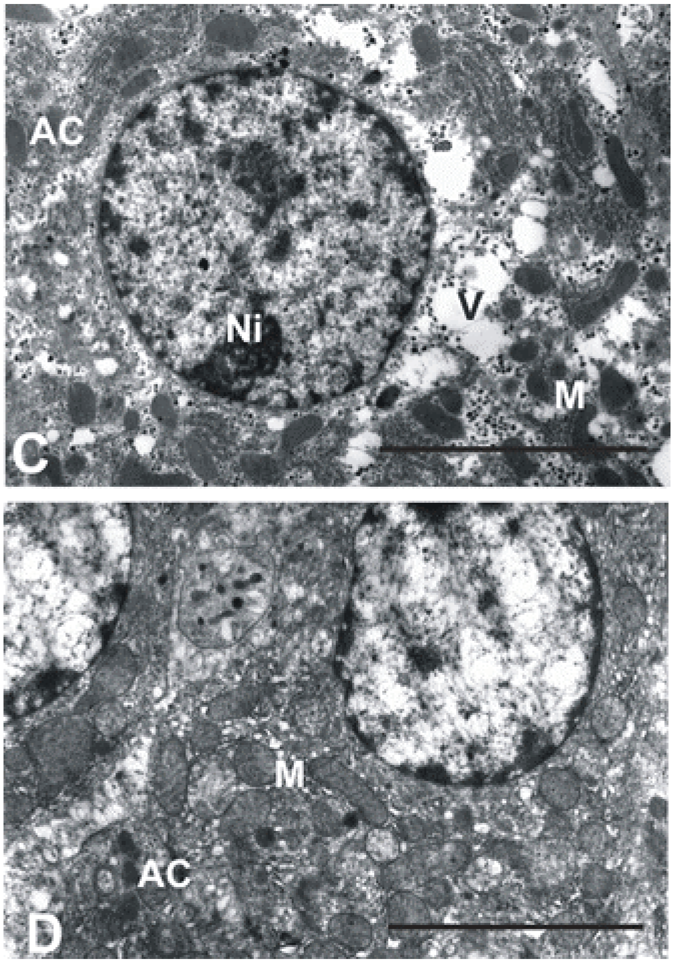

**FIGURE 4C,D**

In 4 h after of cycloheximide administration, alongside with apoptotic hepatocytes described above, we observed cells with nuclei that underwent karyorrhexis (Fig. 5B) and also nuclei with a higher degree of chromatin margination, a specific feature of apoptosis (Fig. 5C).The cytoplasm of these cells is disorganized to the maximum and contains solitary damaged mitochondria, a small number of vesicles, free ribosomes and polysomes, a large number of debris of different organelles, and long, sometimes closed structures, the identification of which is quite difficult (Fig. 5B,C). In liver tissue of young rats, apoptotic bodies containing nuclear fragments are observed (Fig. 5D).

### DISCUSSION

Using the methods of semi- and ultrathin sections, the study of physiological and induced apoptosis in parenchymal cells of the liver contributes to the determination of the early contact stage of apoptosis, both under physiological conditions and cycloheximide impact. In the liver tissue of intact embryos and young rats as well, apoptotic cells are diffused as clusters or groups of two to four cells (Fig. 1). According to other authors[30], the occurrence of apoptotic clusters may reflect either synchronous entry of a group of cells into apoptosis or killing of viable neighboring cells by primary apoptotic cells, propagating the cell death signals via gap junctions.

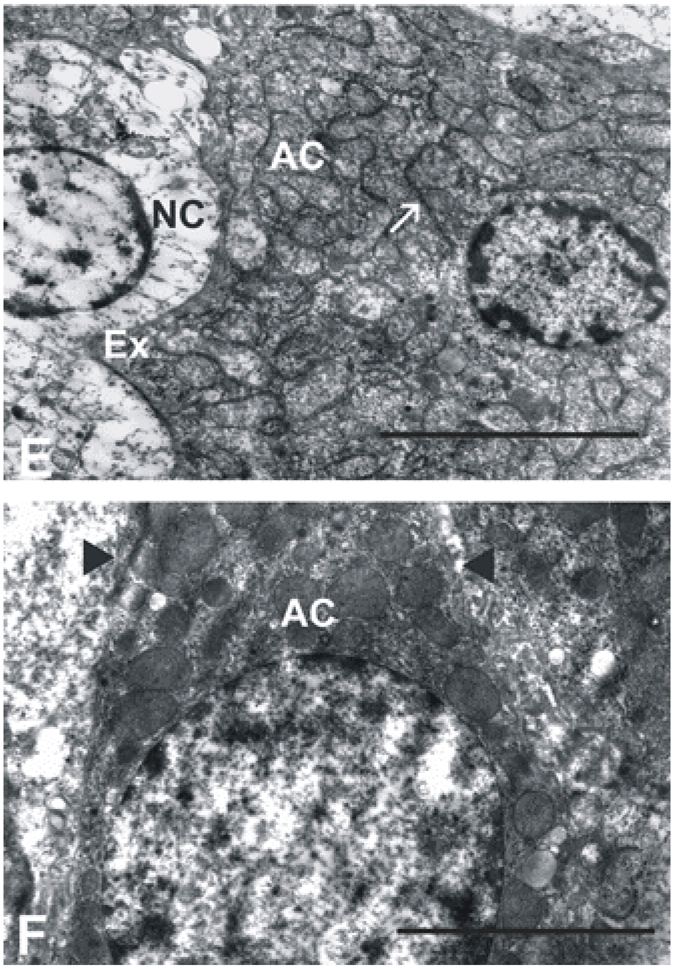

**FIGURE 4E,F**

The presented micrographs (Figs. 1–5) demonstrate that the earliest revealed destruction, both in hepatoblasts and in hepatocytes, is the agglutination of bound ribosomes, reduction of organelles connected with protein synthesis, formation of cytoplasmic excrescences, and changes in cell shape. These processes in apoptotic cells proceed under the steady contact of dying cells with differentiating and/or mitotic hepatoblasts. We suppose that during the contact stage of apoptosis, loss of the initial rounded form of hepatoblasts and polygonal shape of hepatocytes is caused by the breakup of protein synthesis responsible for the rigidity of the cytoskeleton, which stipulates the disorganization and dilution of cytoplasm and its flowing into the formed excrescences. This assumption is confirmed by the data, according to which in apoptosis, the cleavage of gelsolin (actin-modulated protein regulating cytoskeleton)[31] and proteolytic activation PAK2 (p21-activated kinase 2) contributing to the regulation of morphological changes, are observed[32]. Bushell et al.[33] also observed that the levels of both actin mRNA and rpS6 (ribosomal protein S6) mRNA were reduced by approximately 70% (relatively to the control) 2 h after the exposure of Hela cells to TRAIL (tumor necrosis factor related apoptosis-inducing ligand).

According to biochemical data[28,29,34] the overexpression of genes *c-myc*, *c-fos,* and *c-jun* can be observed in the rat hepatocytes immediately (0.5–3 h) after the cycloheximide (an inhibitor of translation and an inductor of apoptosis as well) injection. These genes have both mitogenic[35,36] and proapoptogenic[37,38] features. Hence, the morphological changes in hepatocytes of young rats (Figs. 4 and 5) caused by the inhibition of protein synthesis may also reflect the process of apoptosis induced by

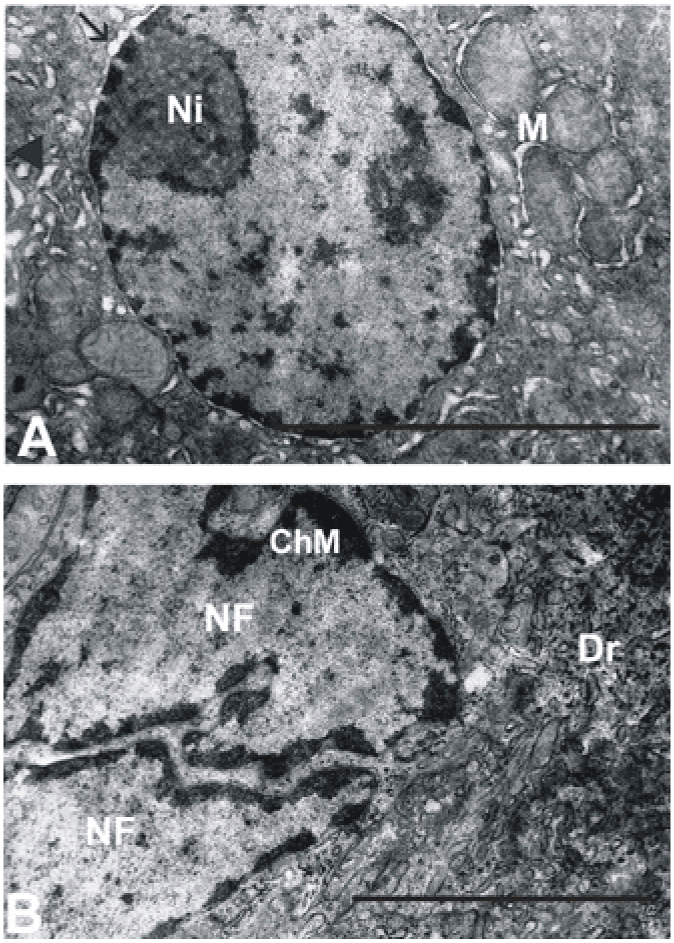

**FIGURE 5.** Ultrastructural changes of hepatocytes of young rats in the process of apoptosis. (A) Large nucleolus (Ni) and primary features of chromatin margination in nucleus with expanded perinuclear space (arrow); swollen cisterns of RER (arrowhead) surrounding mitochondria (M). Three hours after injection of cycloheximide. (B) Apoptotic hepatocyte the nucleus of which underwent karyorrhexis; nuclear fragments (NF) with marginated chromatin (ChM); cytoplasm contains hardly identified structures and debris (Dr) of incompletely dissolved organelles. Four hours after injection of antibiotic. (C) Fragment of nucleus of apoptotic hepatocyte with intensive chromatin margination; cytoplasm contains long, flattened, closed-at-times unidentified elements, possibly debris of mitochondria; solitary empty mitochondria (M). Four hours after injection. (D) Apoptotic body (AB) with great number of nuclear fragments is surrounded by phagocytic cell (PhC). Four hours after injection of cycloheximide. Scale bars 5 µm.

the overexpression of these proto-oncogenes. As such morphological features take place in apoptotic hepatoblasts of fetal liver, we can assume that apoptosis in hepatoblasts is also initiated by the suppression of protein synthesis. The fact that the disturbance of protein synthesis triggers apoptosis is confirmed by data of some authors[8,16], according to which one of the main points of integration of proapoptotic signals can also be the ER. It is established that the ER stress is associated with the accumulation of unfolded or misfolded proteins, i.e., breakup of normal protein synthesis can stimulate the cascade of reactions activating the procaspase-12 that triggers the apoptosis[8]. At the same time, it is also known that the ER stress can also trigger a coordinated adaptive program, UPR, which leads to the inhibition of global protein synthesis, and the selective transcription and translation of proteins, which helps the cell to deal with ER stress[16].

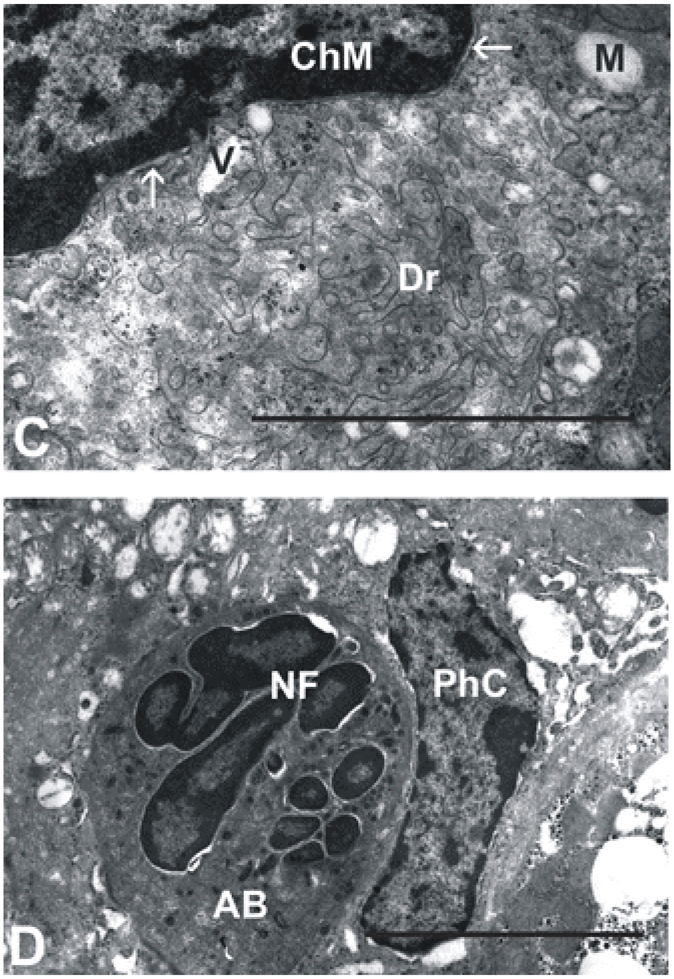

**FIGURE 5C,D**

The ultrastructural study of physiological and cycloheximide-induced apoptosis contributes to the revealing of certain regularity in hepatoblasts and hepatocytes at the contact stage of apoptosis. The triggering of apoptosis in hepatoblasts is accompanied by the decrease of the number of free ribosomes and agglutination of bound polysomes (Fig. 2), and in hepatocytes, the initiation of apoptosis, alongside the decrease of the number of free polysomes, causes significant reduction of elements of granular reticulum and Golgi apparatus, and the agglutination of ribosomes on the remaining cisterns of RER (Fig. 4B–E). At certain stages of apoptosis, in the nuclei of both hepatoblasts and hepatocytes, the nucleolus is eliminated and, accordingly, in the cytoplasm, the reduction of free polysomes is observed (Fig. 2B–D and 4D–F). On the basis of our morphological data, irrespective of the degree of cell differentiation, we suppose that the earliest response of the cell on the induction of apoptosis is the breakup of protein synthesis in apoptotic cells expressed in the reduction of free and bound ribosomes, and in the agglutination of polysomes on remaining elements of RER. The data we obtained, to a certain extent, are consistent with those of different authors, according to which the induction of apoptosis leads to a rapid and substantial inhibition of protein synthesis, and it is accompanied by the changes of the multiple components including translation initiation factors, the ribosome, and cellular level of mRNA[13,14,15,16,33].

At a certain phase of the contact stage of apoptosis, the nuclear shape of hepatoblasts and hepatocytes gradually changes: invaginations of different size appear, the number of chromatin clumps increases, the nucleolus is revealed again, and the number of free ribosomes and polysomes in the cytoplasm increases (Figs. 2E,F and 5A). The reappearance of the nucleolus and the increase of free polysomes can be hypothetically explained in the following way: The agglutination of bound polysomes, i.e., breakup of protein synthesis, causes fault of nuclear-plasma relations in apoptotic cell. The nucleus, which does not receive signals from the cytoplasm, is inactivated gradually, which may result in the elimination of the nucleolus and reduction of free polysomes. According to Todorov[28], in order to maintain its own needs, some organelles in the cytoplasm cleave and, during the estimated time, the cell may use the products of protein and nonprotein splitting. With the exhaustion of cleaved products and adaptation to the new microconditions, the cell begins fighting for its survival. In the 1960s, Campbell et al.[39] established that the free ribosomes, bound to the respective mRNA, might form polysomes on which the protein for the internal use may be synthesized. According to Todorov[28], after the cycloheximide impact, the cell and the cytoplasm contain a certain reserve amount of free ribosomes and mRNA, which represent ready and readily available reserve forms for the creation of new protein-synthesizing polysomes. Based on this fact, we assume that during the survival process, despite the significant (90–95%) inhibition of protein synthesis on bound, but not free, ribosomes[27,28,29], the existence of the viable nucleus in the cell (that is confirmed by its ultrastructure; Figs. 1, 2B–D, and 4B–D) might contribute to the reappearance of the nucleolus and the increase of the number of free ribosomes and polysomes in the cytoplasm (Figs. 2E,F, 4E, and 5A) on which the proteins, necessary for the internal use by the destructive cells, might have been synthesized. Moreover, the data of Holcik and Sonenberg[16], according to which the stress-induced attenuation of global translation is often accompanied by a switch to the selective translation of proteins that are required for cell survival under stress and commonly occurs during cellular stress and apoptosis, allow us to suppose that, possibly, on the very stage of nucleolus reappearance and increase in the number of free ribosomes and polysomes, as a result of reprogramming[28] or selective[16] translation, new proteins could have been synthesized in apoptotic cells on the newly formed free polysomes. The ability of apoptotic cells to synthesize new RNA was noted by other authors as well[40,41,42].

At the same time, one should note that the authors[27,28,29] who were studying the prolonged (0–70 h) impact of cycloheximide on the rat liver hepatocytes discovered that the limited-in-time protein synthesis inhibition is replaced by its reappearance and stimulation, preceding the activation of DNA replication, taking place in the hepatocytes in 60–70 h after the injection of cycloheximide. As we studied the liver tissues during several hours after cycloheximide injection, and this time is not enough for the unambiguous prediction of the subsequent fate of hepatocytes (i.e., determine which cells will die and which will survive), we cannot fully rule out that some morphological alterations, in particular those concerning ribosomes, may be a direct consequence of the cycloheximide treatment.

Further agglutination of free polysomes, intensive expansion of cisterns of RER and perinuclear space (Figs. 2E,F and 5A), chromatin margination, increase of the degree of cell condensation accompanied by the change in its shape, and reduction in its size (as a result of splitting of terminals) indicate the dramatic events preceding the first discrete stage of apoptosis (Figs. 2, 3, and 4D–F). Fragmentation of nuclei and formation of apoptotic bodies, to some extent, proceeds similarly in hepatoblasts and in hepatocytes.

Although the cytoplasm of hepatoblasts undergoes significant changes at the early stage of apoptosis, at the end of this stage degraded organelles are still identified in it, and the mitochondria are often represented as intact forms even in terminals of cytoplasmic excrescences (Fig. 3C–F).

An absolutely different picture is observed in apoptotic hepatocytes, the cytoplasm of which is characterized by a rough degraded structure and it contains small polymorphic vesicles, a great number of incompletely cleaved debris of different organelles, flattened unidentified structures, and solitary destructive mitochondria (Fig. 5B,C). It is possible that such degradation of organelles of the hepatocytes is caused by the acute $Ca^{2+}$ release from the cisterns of RER too. This alters mitochondrial membrane permeability[8] and causes both the release of biomolecules, potentially lethal for the cell (such as cytochrome *c*; caspases 9, 2, and 3; and AIF [apoptosis inducing factor]), from intermembrane space (IMS)[5,43], and subsequent mitochondrial fission, conditioned by the recruitment and activation of DRP1 (dynamin-related protein-1) at discrete sites on the mitochondrial tubular network[44].

According to the results obtained, the morphology of the early contact stage of apoptosis in both investigated objects is similar to a certain approximation. It can be assumed that in other types of cells, the morphological features of the early stage of apoptosis, triggered by the inhibition of protein synthesis, will be similar to the described above.

In this respect, it is interesting to mention the results of Harmon[21], who revealed "dark cell" (the third morphological type of cell death as the author considers) in mice on the bordering zone with induced mastocytoma, and the results of Turmaine et al.[22], who observed cells with characteristic features of "dark degeneration" (a nonapoptotic form of neurodegeneration as the authors consider) in different zones of transgenic mice brain. By their morphological features, both "dark cell" and "dark cell degeneration" are similar to the apoptotic cells we have described above, and they are in close contact with adjacent cells as well. On the basis of this, we suppose that "dark cell" and "dark cell degeneration" are cells at the contact stage of apoptosis induced by the inhibition of protein synthesis. However, it is also possible that the contact stage of apoptosis is typical of only long-living and polyfunctional cells *in vivo*, and the cells in culture or short-living cells either are deprived of this stage or the latter is so short that it is quite difficult to reveal it. This supposition is confirmed by the data of Jacobson et al.[45], according to which in staurosporine-induced apoptosis in GM701 cells, within minutes of treatment, cells change their shape, become stellate, with long thin processes viewed by time-lapse video microscopy.

Hence, we speculate that the data about the nonapoptotic alternative forms of PCD may be due to the absence of morphological characteristics of the early stage of apoptosis, the identification and ultrastructural peculiarities of which are specific and depend on different factors including the cell type, the stage of the development of individual, the time after initiation of apoptosis, and also the stimulus triggering apoptosis and the signaling pathways of its regulation. This can be confirmed by our unpublished data obtained while studying apoptosis in rat hepatocytes induced by dexamethasone under the effect of which the formation of cytoplasmic excrescences and significant change in cell shape of apoptotic hepatocytes are not observed, and by the supposition of other authors according to which the presence of nonapoptotic alternative forms of cell death is mainly conditioned by the cell type rather than by any basic cellular mechanism[46].

We think that the most important and noteworthy fact revealed in our studies is a close contact of apoptotic cells with mitotic and differentiating hepatoblasts (Figs. 1a–d; 2B–F, 3). We suppose that such a coarrangement of cells of the opposite status apparently is not accidental and may reflect certain functional interactions between them. In Fig. 3D, there are presented the contacts between the apoptotic and neighboring cells different in their ultrastructure. The functional significance of the disruption of integrity of plasma membrane between apoptotic and adjoining cells (Fig. 3D*) is unknown to us.

Underneath the membranes of apoptotic and adjoining cells, the presence of formed and forming micropinocytic vesicles and sometimes the localization of vesicles in the space between the apoptotic and adjacent cells (Fig. 3D**) indicate functional interactions between apoptotic and neighboring cells realized through micropinocytosis. Even more, it is possible that micropinocytosis is one of the ways of the utilization of products of proteolytic cleavage, the result of which released amino acids and peptides can be transported from apoptotic cells to the neighboring hepatoblasts, thus contributing to the process of their vital functions. The important role of the proteolytic cleavage processes of the cytoplasmic organelles and micropinocytosis in the functional activity of neighboring cells was noted by Policard and Bessis[47].

In the regions of close contact of apoptotic and neighboring cells, the aggregate of plasma membranes are presented as three dense staining layers (where chains of granules of different size are distributed) alternating with two light ones (Fig. 3D***). By their ultrastructure, they are not identical with the gap junctions observed between the hepatoblasts in fetal liver[48] and in granulosa cells from ovarian follicles of adult Japanese quail[30], but are somehow similar to them. From the literature, it is known that gap junctions, progressively increasing from the $14^{th}$ to the last embryonic day, are observed between the hepatocytes of fetal liver of rats[48], and these junctions are formed by the connexins Cx32 and Cx26[49]. At the same time, there are no data about gap junctional intercellular communications (GJIC) between apoptotic and fetal hepatoblasts. As our morphological results are not confirmed by the experiments with fluorescent markers, specific antibodies to any connexin, gene mutations, or freeze-fracturing, we cannot claim that GJIC are revealed in regions of close contact of apoptotic and mitotic cells.

According to the existing conception, it is necessary that mitosis get balanced by apoptosis for the regulation of cellular homeostasis. The presence of apoptosis-mitosis complex in fetal liver requires an interpretation in the direction of establishment of cause-effect relations in its formation. As was noted before, proto-oncogenes *c-myc*, *c-fos,* and *c-jun* have both mitogenic and proapoptogenic features. The expression of these proto-oncogenes, possibly stimulated through the maternal blood circulation, can trigger both apoptosis and mitosis in neighboring hepatoblasts of fetal liver, depending on the commitment of cells to either of the processes. This assumption, to a certain extent, is based on literary data in which the issue of the existence of common features between the mitosis and programmed cell death is being discussed. According to Jacobson et al.[45], there is increasing evidence that the control mechanisms that regulate the cell cycle and those that regulate PCD share some components. Depending on the conditions, for example, *c-myc* transcript can activate either cell proliferation or PCD, and p53 can either arrest the cell cycle or activate PCD. According to Wyllie[50], one attractive explanation of the finding that *c-myc* have expressed both in proliferating and dying cells is that *c-myc* induce a state in which both cell proliferation and apoptosis become possible, the critical choice between them depending on additional considerations, such as the availability of growth factors, and their relative quantities being determined by the microenvironment.

In this connection, we may assume that the close coarrangement of mitotic and apoptotic cells (Figs. 1b–d and 3A–C) is conditioned by the simultaneous triggering of apoptosis and mitosis in adjacent cells. In this case, as is seen on micrographs presented, the time necessary for the realization of apoptosis (from its initiation to the formation of apoptotic bodies) must be longer than that of the cellular cycle because mitosis in the cell adjacent to the apoptotic cell is practically completed, and the apoptotic cell is still at the contact stage and needs additional time to be fragmented into apoptotic bodies. During this additional time, the neighboring mitotic cell divides and daughter hepatoblasts will already be in contact with the apoptotic cell. Therefore, in case of the simultaneous initiation of apoptosis and mitosis in adjacent cells, the apoptosis-mitosis complex will not be observed in fetal liver. Furthermore, apoptosis-mitosis complex will not be observed in the case when the initiation of the mitosis in the neighboring cells precedes apoptosis.

In our opinion, the apoptosis-mitosis complex can be formed only in the case when apoptosis precedes the mitosis in neighboring cells. This is confirmed by the results that we obtained when studying apoptosis in the early stage of rat liver regeneration after the partial hepatectomy under normal conditions and after the impact of chloramphenicol an the apoptosis inhibitor. According to these results, the apoptosis revealed in the remnant liver shortly after the hepatectomy precedes both the DNA synthesis and mitosis, and plays an important role in the liver regeneration (unpublished data).

According to the literary data, the induction of apoptosis is associated with a rapid and substantial inhibition of protein synthesis that is accompanied by the changes of the multiple components, including translation initiation factors, the ribosome, and cellular level of mRNA[13,14,15,16]; one can assume that the early contact stage of apoptosis is accompanied by the complex structural and functional changes. It is natural to assume that the structural and ultrastructural changes, which are present in the cells of both of the objects that we studied (Figs. 1–5), are caused by the functional disorders, which, according to the data of others authors[16,28,33], are often accompanied by the switching to the selective[16] or reprogrammed[28] translation, and transcription of the proteins that is necessary for the cells' survival under the conditions of stress and apoptosis. Based on this, we suppose that in the early stage of apoptosis, triggered by the disorder in the protein translation, the synthesis of new proteins and RNA is possible, which also conforms to the data of other authors[28,29,40,41,42]. Our assumption, to a certain extent, is confirmed by the data of Bushell et al.[33], who assume that the changes observed in early apoptosis lead to the specific translational repression of a large group of the mRNAs, but allow continued or enhanced translation of other mRNAs. Based on the afore-mentioned, we speculate that at an early stage of apoptosis through selective transcription and translation, low-molecular substances may be synthesized in the cells that, under the optimal conditions, will be able to diffuse from the apoptotic cell into the neighboring one by micropinocytosis and possibly through the gap junctions.

Trosko and Ruch[51], while considering the role of intercellular communications in carcinogenesis, give a model of Loewenstein published in 1972. According to this model, cells are able to produce negative and positive low-molecular-weight signals that will be diffused in adjacent cells through the gap junctions; the negative signals would inhibit cell division and maintain differentiation in neighboring cells, whereas the positive signals would stimulate cell growth and prevent differentiation. We suppose that this model can work to some extent even in the case of contact of apoptotic cells with the hepatoblast and hepatocyte. Our supposition that the connection between apoptotic cells and the hepatoblasts and hepatocytes bear a functional character was confirmed by results of other authors that established that early apoptotic cells (until the loss by the dying cells of their plasma membrane integrity) were still coupled with healthy cells through gap junctions, the number of which was decreasing during the process of apoptosis[52,53]. In their review, Krysko et al.[30] discussed the data on communications between dying and healthy cells, and supported the hypothesis that gap junction channels may propagate cell death and survival modulating signals.

On the basis of our own analysis and literature data, we suppose that the apoptotic cell may contribute to the multicomponent process of proliferation of neighboring cells as a result of activation of proteolysis and synthesis of new low-molecular substances, and that apoptosis precedes mitosis in the formation of close contact between apoptosis and mitosis (Figs. 1b–d, 2E, and 3A–C). Hence, one might assume that in order to maintain the cellular homeostasis in the tissue, the death of one cell must stimulate the “birth” of another. This assumption is confirmed by Kondo’s[54] data, according to which cell death can be described as programmed or altruistic for the benefit of an organism as a whole if their suicide stimulates proliferation of healthy cells to replace them. Policard and Bessis[47] underlined the important role of proteolytic cleavage in cells in tissue regeneration and regulation of mitosis as far back as the last century. They related this to a long-standing opinion that the death of one cell causes the formation of another.

Based on the above, we can assume that apoptosis induced by the inhibition of protein synthesis, both in hepatoblasts and in hepatocytes, represents a multistage process that, alongside the first discrete stage and the stage of utilization of apoptotic bodies[1], also includes the early contact stage. It continues from the moment of cell death initiation until the separation of the apoptotic cell from its neighbors. The features of the early stage of apoptosis are the agglutination of bound ribosomes, the reduction of cisterns of RER in differentiated or differentiating cells, the dissolution of the nucleolus, the reduction of free polysomes, the formation of cytoplasmic excrescences, and the changes in cell shape. In a certain phase of the early stage of apoptosis, the nucleolus is revealed in the nucleus again and the number of free polysomes in cytoplasm increases, which, according to other authors, suggests the synthesis of new RNA and proteins[28,29,40,41,42]. We hypothesize that the apoptotic cell, alongside the products of proteolytic cleavage, can contain newly synthesized, low-molecular substances, which may contribute to both functional activity and proliferation of neighboring hepatoblasts by micropinocytosis and possibly through the gap junctions. Therefore, the function of apoptosis may not be not limited only to the elimination of harmful, damaged, and unwanted cells, and this changes the existing view about the function of apoptosis and its role in maintaining cellular homeostasis.

### ACKNOWLEDGMENTS

We would thank Dr. Nugzar Bakradze for the active technical assistance. We are also grateful to all authors whose research contributed to a better understanding of our results.
